# “It Don’t Mean a Thing if It Ain’t Got that Swing”– an Alternative Concept for Understanding the Evolution of Dance and Music in Human Beings

**DOI:** 10.3389/fnhum.2016.00485

**Published:** 2016-10-07

**Authors:** Joachim Richter, Roya Ostovar

**Affiliations:** Institute of Tropical Medicine and International Health, Charité UniversitätsmedizinBerlin, Germany

**Keywords:** beat, rhythm, dance, embodied music cognition, embodied communication, rocking, human universals, human evolution

## Abstract

The functions of dance and music in human evolution are a mystery. Current research on the evolution of music has mainly focused on its melodic attribute which would have evolved alongside (proto-)language. Instead, we propose an alternative conceptual framework which focuses on the co-evolution of rhythm and dance (R&D) as intertwined aspects of a multimodal phenomenon characterized by the unity of action and perception. Reviewing the current literature from this viewpoint we propose the hypothesis that R&D have co-evolved long before other musical attributes and (proto-)language. Our view is supported by increasing experimental evidence particularly in infants and children: beat is perceived and anticipated already by newborns and rhythm perception depends on body movement. Infants and toddlers spontaneously move to a rhythm irrespective of their cultural background. The impulse to dance may have been prepared by the susceptibility of infants to be soothed by rocking. Conceivable evolutionary functions of R&D include sexual attraction and transmission of mating signals. Social functions include bonding, synchronization of many individuals, appeasement of hostile individuals, and pre- and extra-verbal communication enabling embodied individual and collective memorizing. In many cultures R&D are used for entering trance, a base for shamanism and early religions. Individual benefits of R&D include improvement of body coordination, as well as painkilling, anti-depressive, and anti-boredom effects. Rhythm most likely paved the way for human speech as supported by studies confirming the overlaps between cognitive and neural resources recruited for language and rhythm. In addition, dance encompasses visual and gestural communication. In future studies attention should be paid to which attribute of music is focused on and that the close mutual relation between R&D is taken into account. The possible evolutionary functions of dance deserve more attention.

## The Evolution of Dance and Music, Current Concepts

The origin of dance and music, beautiful and powerful universals of humankind is a mystery. All over the world there are myths on how humankind received dance and music. In Hindu mythology, the god Shiva Nataraj created the world by dancing. In most traditional cultures dance plays a pivotal role (Métraux, 1958; Lapassade, 1976; Verger, 1982; Ginn, 1990; Christoph and Oberländer, 1996; Foix, 2007; Kouam and Mofor, 2011).

In the Western world attention has been usually paid to the origin of language and to its relation to the melodic attribute of music whereas dance and rhythm have been, for long, neglected. This might partly be an unintentional consequence of the duality of body and mind concept of Cartesian philosophy as well as the historical hostility of the Roman Catholic and Protestant Churches toward dance. This had led to the omission of percussion instruments in European classical music, thus, diverting the first attention from rhythm and dance to melody (Redmond, 1997; Wagner, 1997; Foix, 2007).

The evolution of music has become an important research topic rather recently (Falk, 2000; Cross, 2005; McDermott and Hauser, 2005; Mithen, 2007; Cross and Morley, 2008; Dissanayake, 2008; Patel, 2008; Patel et al., 2008; Tomasello, 2008; Panksepp, 2009; Merker et al., 2015).

The evolution of music has become an important research topic rather recently. The interest is partly awakened by neuroscience, basically to identify the core components of human cognition (Oberzaucher and Grammer, 2008; Thaut et al., 2008; Costa-Faidella et al., 2011; Nozaradan et al., 2011a,b, 2012; Alluri et al., 2012; Arnal and Giraud, 2012; De Guio et al., 2012; Teki et al., 2012; Leman and Maes, 2014) in comparison to animals (Fitch, 2006, 2012, 2013; Ravignani et al., 2013, 2014). The evolutionary role of dance is even more enigmatic than that of music considering that who dances dispenses considerably more energy than a singer or a musician. The evolutionary functions of dance have received more attention only recently (Dean et al., 2009; Hanna, 2010; Whitehead, 2010; Grammer et al., 2011; Neave et al., 2011; Davidson and Emberly, 2012; Fitch, 2012; Christensen et al., 2014; Morley, 2014; Woolhouse and Lai, 2014; Wang, 2015).

### Definition of Dance

Neither the term dance nor the term music as such are precise. “Dance” in Oxford’s dictionary is defined as: “move rhythmically to music, typically following a set sequence of steps.” For our purpose we define as “dance” body movements coordinated to a basic rhythm. *Rhythm* is constituted by a *pulse* or sequence of *beats* which are organized hierarchically. There are four main sub-constituent elements of rhythm: (1) *tactus* represents identical short-duration periods subdivided into strong beats (“downbeats”) and weaker beats (“offbeats”); (2) *tempo:* the frequency of the tactus; (3) *meter:* cyclical groupings of beats into units marked by accents; (4) *patterns:* sequences of time intervals that may or may not extend across meter units (Fitch, 2013; Thaut et al., 2014). Dance differs from simple synchronization to a simple regular pulse, because dance offers the possibility to vary steps with respect to beats inside the tactus. Nevertheless, the dancer has to respect a basic groove (Janata et al., 2012; Fitch, 2013; Oota, 2016). We would therefore not consider soldiers marching or harvesters working in synchrony with a beat as dancers: they do not differentiate down- and offbeats of a rhythm and they have a defined purpose. We also see modern non-rhythmic expressive dance as theater rather than dance. We would also not describe as dance the repeated steps without keeping a regular basic pulse as described for some birds (Ota et al., 2015).

On the other hand, we extend our definition of dance to the beat-keeping movements of music performers. Embodied perception is the physiological fundament of this phenomenon. Unintentional body movements to a beat reflect the role of our body in rhythm perception. For dancing, the capacity of beat anticipation and of embodied rhythm perception are required (Phillips-Silver and Trainor, 2005; Honing, 2012; Bouwer et al., 2014).

### Conceptions of Music and Theories on the Origin of Music

“Music” and “dance” encompass overlapping spectral and temporal attributes. Spectral attributes are pitch, intervals, and harmony. Temporal attributes are covered by rhythm consisting in its sub-constituent elements tactus, meter, tempo, and pattern (Fitch, 2013; Janata and Parsons, 2013; Thaut et al., 2014; Lee et al., 2015; **Table 1**). Loudness and dynamics are, albeit important, not specific for music since these are also expressive means of other arts such as theater, poetry, rhetoric or cinema. For music we discern its three main specific attributes: *rhythm, melody*, and *harmony*. Rhythm is music’s central organizing structure. Rhythm is indispensable for both, dance, and music (Honing, 2012; Thaut et al., 2014). Whereas, rhythm can exist without melody or harmony, melody or harmony cannot exist without rhythm. The concepts of melody and harmony are partly defined by their temporal rhythmic fundament: melody is defined as a series of sounds with a different pitch over time. Harmony must be subdivided into “*sequential harmony*” which means the defined pitch intervals in a melodic time line and “*polyphony*” which means simultaneous sounds with different pitches following different melodic lines. Authors of publications on the evolution of music, usually do not mention which of these attributes are precisely meant. Usually, only the melodic and to a lesser extent the harmonic attribute of music have been focused on. On the other hand, rhythm has been relatively neglected. Also in the past, scholars who reflected on the origin of music referred to its melodic attribute. The philosopher Rousseau (1781) argued that ancestral humans would have used a proto-musilanguage and that people would have communicated by singing. The German poet Heine (1822) interpreted melodic music as a precursor of language. Darwin (1871, p. 572) in “The descent of man” noted: “as neither the enjoyment nor the capacity of producing musical notes are faculties of the least use to man in reference to his daily habits of life, they must be ranked amongst the most mysterious with which he is endowed.”

**Table 1:** Relations and overlap between dance and music attributes.

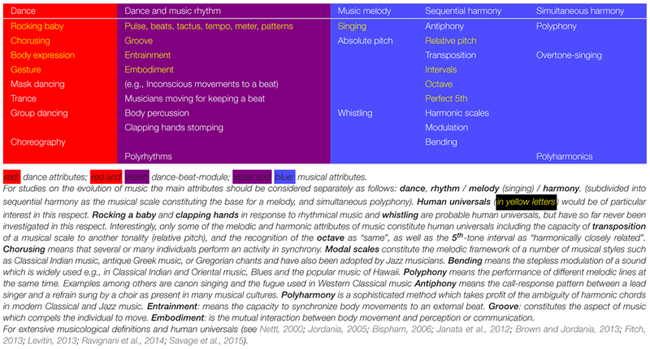

Most anthropological, psychological, and musicological references focus on the evolution of melodic music but not of rhythm nor of dance. A favorite theory on the evolution of music has been that it would have evolved just as a by-product of language evolution (Pinker, 1997; Jourdain, 2001; Brown and Jordania, 2013).

### Current Hypotheses on the Evolution of Dance

Would also dance constitute a mere by-product of language evolution? It has been proposed that the capacity to move in time with an auditory pulse, i.e., entrainment would have evolved as a by-product of vocal mimicry (Schachner, 2013). We believe that R&D are not mere evolutionary by-products of language. Scientific studies showed that infants are able to extract and anticipate a rhythmical pulse and that they have a strong impulse to move spontaneously when exposed to an external rhythm (Hannon and Johnson, 2005; Hannon and Trehub, 2005; Phillips-Silver and Trainor, 2005; Winkler et al., 2009; Fujii et al., 2014). We hypothesize that R&D are part of an inborn series of physiological reflexes universal in all humans. The observation that children at a certain age inevitably dance when they are exposed to a rhythm has been supported by a cross-cultural questionnaire study conducted in three continents by our group (Ostovar, 2016). If such a reflex was constant and genetically determined, it could join the list of physiological reflexes used in developmental psychology and pediatric neurology. The confirmation of the existence of such an innate reflex would also support the concept that dance have had a considerable importance in the evolution of humankind.

This prompted us to perform a literature search in the fields of medicine and developmental psychology, philosophy, archeology, anthropology, ethnology, and musicology focusing on the evolution of dance in both, children and in humankind. We were surprised about the scarcity of references. Neither in textbooks of pediatrics nor of developmental psychology a physiological dance reflex or dance reaction to a beat occurring during infantile development is mentioned (Ringwalt, 2008; Berk, 2014). A PubMed search on “dance reflex” yielded a total of 22 hits, of which none dealt with our topic.

### Music and Dance as Pre-verbal Communication Tools

What could be the evolutionary functions of rhythm and dance? One has to bear in mind that people who dance, stomp, and clap hands are noisy and less aware of predators (Hart and Sussmann, 2009). Such a behavior must not, at least, have had survival disadvantages if it was to be conserved in evolution.

Donald (2001) formulated the theory that long before the evolution of language humans were communicating by extra-verbal means which he called mimesis. Mimesis can be imagined as how we communicate in a foreign country without any knowledge of the local language. This theory was later taken up by Mithen (2007) who called extra-verbal communication the “hmmmm”-communication (hmmmm stands for holistic multi-modal manipulative musical). In this context Mithen (2007) argued that music would have been a means of pre-verbal communication, calling his book “The Singing Neanderthals.” This title reflects the wide-spread opinion that melody is the main attribute which characterizes music.

Since we believe that rhythm is the fundament of music we would propose for the next book the title “The *Swinging* Neanderthals.” Masataka (2009) sustains the view that both language and music evolved from a pre-linguistic communication system which was neither language nor music. Similarly, Brown (2007) proposes that music has evolved from a pre-linguistic precursor present also in animals which he called “contagious heterophony.” An alternative account is proposed by Livingstone and Thompson (2009) where music originated from a more general adaptation known as the “Theory of Mind” which would allow an individual to recognize the mental and emotional state of conspecifics. Underpinned by the mirror neuron system of empathy and imitation, music would achieve engagement by drawing from pre-existing functions across multiple modalities (Rizzolatti et al., 1996; Wu et al., 2016). This, in our opinion, applies even more to dance because of its strong interaction between perception and motor response. Whitehead (2010) puts forward the hypothesis that song and dance would have even preceded mimesis in hominid evolution. Since most authors looked for the origin of music in melody and singing, some of them interpreted as their precursors the interaction between pre-musical utterances of the infant by modulation of crying and melodic modulation of language by the caretaker, also called “motherese.” The origins of “motherese” would already be established during prenatal development of the fetus (Parncutt, 2009). “Motherese” has been proposed as proto-melody paving the way for the evolution of music. Rhythm in this respect has not been considered (Dissanayake, 2004; Falk, 2005; Hagen and Hammerstein, 2009; Panksepp, 2009; Wermke and Mende, 2009; Trevarthen, 2011; Brown and Jordania, 2013). Furthermore, although all mothers of all cultures and all ages know that babies are soothed by being rocked, this is, if ever, only exceptionally mentioned and has also never been proposed as a human universal (Brown, 1991; Antweiler, 2015).

Summarizing, the functions of R&D as pre- and powerful extraverbal communication tools have found little attention.

## Defining A Differentiated Framework of Music and Dance

For defining the evolutionary functions of music a more fine-grained concept is required (Hagen and Bryant, 2003; Fitch, 2006; Levitin, 2013). Dance is usually conceived as being distinct from music. The close relationship between rhythm and dance has been acknowledged only recently (Phillips-Silver and Trainor, 2005; Dean et al., 2009; Phillips-Silver et al., 2010; Janata et al., 2012; Phillips-Silver and Keller, 2012; Stevens, 2012; Morley, 2014). The pivotal importance of rhythm, synchronized body movements and dancing as prerequisites for the emergence of all attributes of music has found relatively little attention. Whereas, rhythm and dance can exist without melody, there is no music without rhythm (Honing, 2012). Music has also been considered for a long time from the viewpoint of a unimodal phenomenon (auditory processing), whereas, it is multimodal (through action–perception coupling; Lesaffre and Leman, 2013). There is no reason to assume that the different attributes of music evolved at the same time and pace. Only two attributes of music constitute human universals: all cultures have rhythm and almost all have melody (songs; **Table 1**). Polyphonic harmony has developed only some 1000s of years ago and has been explored only since historic times starting in Ancient Greece (Jordania, 2005). Among the attributes of melody and sequential harmony only the octave, the perfect fifth and building a melodic scale by the division of the octave into unequal intervals are human universals [Nettl, 2000; Savage et al., 2015 (see also **Table 1**)]. Many cultures such as in the Arabic world, in Turkey, in Persia or in India, have, instead of polyphony, pursued to evolve melody by adding particular intervals and bending melody sounds in a sequential harmonic way (modal music).

On the other hand, in most cultures, worldwide, the concept and terms of R&D are not separated from each other (e.g., the terms “Samba,” “Salsa,” “Guaguancó,” “Tango,” “Waltz” apply to both, to the rhythm and to the dance).

In line with this, we propose an alternative conceptual framework which focuses on the mutual co-evolution of R&D (Phillips-Silver and Trainor, 2005; Panksepp, 2009; Lesaffre and Leman, 2013; Levitin, 2013; Maes and Leman, 2013). In other words, R&D are two sides of the same coin. Not only do we move to what we hear but what we hear depends on how we move (Phillips-Silver and Trainor, 2005). The unintentional body movements when we perceive a “groove” (defined as the aspect of music which compels us to move), among all body parts primarily involve the lower limbs confirming the close relation to dance (Janata et al., 2012). The mutual connection between R&D is also reflected by the musical terms “downbeat” and “offbeat,” where the downbeat indicates the dance step which carries the weight of the body when it comes back down onto the legs.

Movement, rhythm and emotional well-being have particular neural pathways involving cerebellar structures which coordinate sensory neuronal inputs with motoric responses (Molinari et al., 2003; Levitin, 2006; Thaut et al., 2008; Lehmann, 2010; Nozaradan et al., 2011a,b, 2012; Grahn, 2012; Teki et al., 2012; see the comprehensive reviews of Levitin, 2013; Repp and Su, 2013; Chauvigné et al., 2014). Even deaf children have the impulse and the capacity to dance by perceiving the beat through their cutaneous pallesthesic and visual receptors (Phillips-Silver et al., 2015).

There are relatively few references on the specific evolutionary functions of rhythm in humans, mainly in comparison with non-human animals (Honing, 2012; Fitch, 2013; Ravignani et al., 2013, 2014). Rhythm cognition depends on body movement and vice versa (Lesaffre and Leman, 2013; Christensen et al., 2014). Fitch (2013) emphasizes that necessary prerequisites of research on rhythm are unambiguous definitions of the terms “rhythm” and its sub-constituent elements “beat,” “pulse,” “meter,” “tempo,” and “tactus” (**Table 1**). Dancing requires the perception of such hierarchically structured rhythms in order to coordinate and differentiate those steps which carry the whole bodyweight (usually the downbeats) from steps carrying less or no weight (offbeats). Sequences of arbitrary pulses are therefore spontaneously classified into a rhythmical tactus by the dancer, a capacity observed already in infants (Phillips-Silver and Trainor, 2005).

For people of traditional societies the importance of dance and the tight connection between R&D are beyond any doubt (Ginn, 1990; Morley, 2014). In societies with strong dance traditions, singing out of tone is tolerated more easily than drumming only slightly out of beat. This is what the composer Duke Ellington meant with his song: “it don’t mean a thing if it ain’t got that swing.” Instead, passive listening to music without moving, as seen in listeners to Western classical music, requires an educational effort, as it can easily be seen when children are obliged to sit still in a concert. In fact, spontaneous unintentional body movements when listening to a rhythmical beat are difficult to suppress (Molinari et al., 2003; Levitin, 2013).

Moreover, dance is a comprehensive art encompassing attributes which go beyond music such as its external visual signals. The dancer is seen by others acting as a moving picture (Hanna, 2010; Lee et al., 2015). This also applies to a widespread and very old form of dance, i.e., round dance, where the group dances and different dancers enter into the round to perform their solo before rejoining the round as it is also commonly observed in children. Furthermore, dance may encompass gestural and dramatic codes and, thus, has paved the way for the development of theater. The comprehensiveness of dance made already Curt Sachs argue that dance would be the mother of all arts (Sachs, 1933/1980).

## Connecting Studies in Various Scientific Disciplines with Hypotheses on the Evolution of Dance and Music in Humankind

### Evidence of Dance and Music in Archeological Records

When we look for archeological proof for music and dance we must bear in mind that archeology depends on the finding of artifacts indicating the presence of a certain human behavior at a certain time. The earliest artifacts confirming musical activities are around 45,000 years old. Examples are preserved instruments such as flutes in the neolithic caves of “Hohle Fels” and “Geissenklösterle” (Higham et al., 2012). Before, we assume that music and dance did not develop earlier than this, we must acknowledge that in a hunter-trapper-gatherer society not only any artifact constitutes an additional weight to carry but not to leave one’s traces also constitutes part of the survival strategy. Moreover, many instruments are natural objects, such as conch shells, or are made of perishable materials, such as wood and animal skins, which are not preserved for long (Ginn, 1990). Furthermore, humans always possessed a versatile instrument without the necessity of producing a musical instrument, i.e., their own body (Barbagiovanni, 2006). Some examples of human behavior appear so omnipresent and obvious to us that it has, in fact, never been investigated if these are universal in all human societies. One of these is accompanying a beat by clapping hands, a behavior which is observable in humans of all ages (Brown, 1991; Antweiler, 2015; Savage et al., 2015). Another actual example of body percussion and dance is “Flamenco” which in its original form was performed only by singing, clapping hands and stepping on the ground without the use of any musical instruments (Caballero, 1992). This practice is also illustrated by the “Akonhoun” dance tradition of Benin, where dancers perform percussion on their body while dancing as well as “Schuhplattler” in German folk music. To support our view, one may consider in analogy the development of painting, where the human body was the first canvas as confirmed by ca. 200,000-year-old red ochre findings in several places where Neanderthals were living (Wrescher, 1980; Roebroeks et al., 2011). Cave paintings and sculptures confirming dancing are relatively recent. The oldest cave paint possibly representing a dancer is the around 35,000-year-old “magician” (French: “sorcier”) of the “trois frères cave” in Southern France, a zoomorphic figure with animal and human characteristics. Such mask dancing is still practiced in traditional societies aiming at being possessed by an animal spirit (Ginn, 1990; Christoph and Oberländer, 1996). Unequivocal dancing scenes are represented in paintings in the “Valcamonica” and “Addaura” caves in Italy which are no older than 10,000 years (Anati, 1995).

### Studies on Infants and Children

Studies on infants, toddlers, and children contribute to elucidate the evolution of dance and music. The study group of Henkjan Honing showed in a very elegant experiment that newborns already perceive and anticipate musical pulses, a phenomenon which was called “beat induction” (Winkler et al., 2009). Beat processing has been shown to be pre-attentive for metrically simple rhythms with clear accents (Bouwer et al., 2014). Three- to four-month-old infants demonstrate spontaneous limb movements coordinated to a musical pulse (Fujii et al., 2014). Furthermore, infants are able to stratify musical pulses into meters (Phillips-Silver and Trainor, 2005). This capacity is linked with body movement. Infants use meter to categorize rhythms and melodies and learn more readily to tune into musical rhythms than adults (Hannon and Johnson, 2005; Hannon and Trehub, 2005). Human infants spontaneously engage in significantly more rhythmic movement to music and other rhythmically regular sounds than to language (Zentner and Eerola, 2010). The precocity of beat induction already being observed in newborns, the ability to stratify musical beats into tactus and meters, the efficacy of rocking, the unintentional movements to rhythm, the pre-attentive characteristic of beat processing, and the intensity of the emotional impact of R&D on humans, support our view that in human evolution communication through R&D preceded verbal and melodic communication (Bergeson and Trehub, 2005; Winkler et al., 2009; Hagen et al., 2010; Whitehead, 2010; Nozaradan et al., 2011a,b, 2012; Honing, 2012; Lesaffre and Leman, 2013; van Noorden, 2013; Bouwer et al., 2014; Leman and Maes, 2014; Morley, 2014; Ravignani et al., 2014; Miura et al., 2016). Not only environmental but also genetic factors have been shown to play a role in our ability to perceive rhythm (Seesjärvi et al., 2015).

## What Could the Evolutionary Functions of Rhythm and Dance Be?

With this differently defined framework one may reformulate the question “what was the function of music in human evolution?” in a more particularized way: “what was the function of rhythm and dance?”

Did this module pave the way for further evolution of other attributes of music and of language? Future studies may elucidate to what extent R&D are exclusively human or to which capacities in this respect non-human animals are capable. Also some birds and a captive sea lion are able to anticipate beat and move to it to some extent, but apparently animals do not subdivide rhythm into more and less accentuated beats (Patel, 2008; Patel et al., 2008, 2009; Kirschner and Tomasello, 2009; Cook et al., 2013; Ravignani et al., 2013, 2014). Chimpanzees display spontaneous rhythmical behaviors (drumming and carnival display; Arcadi et al., 1998, 2004; Fitch, 2006; de Waal, 2013; Ravignani et al., 2013) and an experiment has shown that a trained chimpanzee was able to anticipate a rhythmical beat (Hattori et al., 2014). Dancing, however, contrary to marching in lockstep or synchronized working to a beat, requires a sophisticated hierarchical perception of rhythm (Fitch, 2013). Therefore it is, in our opinion, very unlikely that dancing simply constitutes a mere by-product of entrainment. What are R&D in humans good for? Why is the impulse to dance so powerful? What could be the evolutionary functions of R&D?

## Reproductive Fitness and Sexual Attractiveness

Contrary to other scholars of his time, Darwin (1871, p. 880) had also the rhythmic aspect of music in mind as well as its reproductive fitness advantage, as he wrote “we may assume that musical tones and rhythm were used by our half-human ancestors, during the season of courtship.” “Dancing is the vertical expression of a horizontal desire” a quote attributed to Robert Frost to which George Bernard Shaw added: “legalized by music,” confirms this observation in poetry. Sexual attractiveness has been since long hypothesized to be the main evolutionary function of dance but has become a scientific research focus only recently (Dissanayake, 2008; Oberzaucher and Grammer, 2008; Dean et al., 2009; Hanna, 2010; Grammer et al., 2011). Rhythmicity has been proposed as an indicator of mate quality (van den Broek and Todd, 2009). Furthermore, dancers are able to communicate subtle non-verbal signals (Oberzaucher and Grammer, 2008; Hanna, 2010; Grammer et al., 2011). The Latin-derived French word “emotion” does not by mere chance contain the word “motion” (Harper, 2001–2016). R&D move us profoundly. Motion alone can effectively communicate emotion, charisma and sex appeal (Oberzaucher and Grammer, 2008).

To say it with the words of Hanna (2010, p. 2): “Dance and sex both use the same instrument — namely, the human body — and both involve the language of the body’s orientation toward pleasure. Thus, dance and sex may be conceived as inseparable even when sexual expression is unintended. The physicality of dance imbued with “magical” power to enchant performer and observer, threatens some people (Wagner, 1997; Karayanni, 2005; Shay and Sellers-Young, 2005). The dancing body is symbolic expression that may embody many notions. Among these are romance, desire, and sexual climax.”

Movement quality not only seems to indicate mate quality, but also the interest of a potential partner, which could denote the probability of successful mating (Grammer et al., 2011; Neave et al., 2011).

## Social Fitness

### Synchronization of Many Individuals

Synchronization is a behavior not limited to humans (Ravignani et al., 2014). It may have a direct effect on predators or reflect the general advantages of cooperation via positive social interactions, a finding also observed in macaques (Nagasaka et al., 2013). Rhythm enables the synchronization of 1000s of dancing human beings such as in a rock concert (Canetti, 1960). The dynamics of rhythmic synchronization differ fundamentally from that of a swarm: a swarm is coordinated by an energy wave passing very quickly but consecutively through many individuals sensing the movement of the adjacent individual. This confounds a predator on which individual prey to catch. Humans, presenting the simultaneous movement of a stomping crowd screaming and armed with fire, may delude a predator by producing the impression of being a homogeneous enormous animal which would be too powerful to attack. This effect may be taken advantage of also in hunting battues (Hagen and Bryant, 2003; Bispham, 2006; Trevarthen, 2011; Phillips-Silver and Keller, 2012; Repp and Su, 2013).

It has recently been argued that self-generated sounds of locomotion and ventilation interfere with the perception of the surroundings. The synchronization of the movement of a number of individuals would thus increase the duration of the intervals where the surroundings can be heard better (Larsson, 2014). This means that synchronization would constitute a by-product of hunting abilities. To prove this hypothesis one would expect that traditional hunters follow animals to hunt in a kind of lockstep, an observation that has not been provided so far.

### Social Bonding

In humans, the synchronic movement leads to “muscular bonding” which enables to overcome emotional boundaries between individuals and, thus, strengthens the community (Wiltermuth and Heath, 2009).

A pivotal fitness strategy of hominids is cooperation (Nowak, 2006; Nowak et al., 2010). Drumming and dancing are profoundly social activities (van Noorden, 2013). Some of the countless examples of this way of social engagement are “Samba de Roda,” “Flamenco,” and Senegalese “Sabar,” where the audience supports the musicians and dancers rhythmically, members of the audience enter into the round for dancing and the drummers and/or other musicians interact directly with the dancer. In many musical cultures the dancer is a percussionist at the same time, as it may be observed not only in traditional societies but also on ancient Egyptian and Greek frescoes or in actual tap-dancing (Caballero, 1992; Redmond, 1997). In many societies dancing is an integral part of important group ceremonies such as initiation rites or weddings. In hunter-gatherer societies, groups may be limited to 40–50 people. The future spouse has to leave her or his group after the wedding in order to join the partner’s group. By dancing, future spouses demonstrate their ability, strength and elegance not only to the future partner but also to other members of the group which will admit the spouse as a new member. In other words, promised spouses need also to catch the eyes of the mothers and fathers in law or other group members who have a say. This latter aspect has, to our knowledge, not yet been explored. Bonding by R&D strengthens the community. Musicians delight dancers. They offer the fundament for the joy of the dancers. This profoundly emotional type of embodied extra-verbal communication increases the group’s cohesion and the identification with the group (Kirschner and Tomasello, 2009, 2010; Boer et al., 2011, 2012, 2013; Davidson and Emberly, 2012; Boer and Abubakar, 2014; Kirschner and Ilari, 2014). Music and especially rhythm constitute a deeply rooted signaling system for extra-verbal communication evoking emotional reactions of other potentially cooperating individuals (Bryant, 2013). The propensity to move in time to rhythmic percussive sounds is manifest from an early age on, as seen in children’s impulsive body movement in response to music (Zentner and Eerola, 2010). Joint drumming facilitates the synchronization in preschool children (Kirschner and Tomasello, 2009). Interpersonal synchrony increases helpfulness already in 14 month-old toddlers and the promotion of prosocial behavior by interpersonal rhythmic synchrony has been confirmed in cross-cultural studies in 4-year-old children as compared to matched controls (Kirschner and Tomasello, 2010; Cirelli et al., 2014; Kirschner and Ilari, 2014; Trainor and Cirelli, 2015). R&D are also potent collective mood synchronizers (Hagen and Bryant, 2003; Wiltermuth and Heath, 2009; van Noorden, 2013). The emotional impact of the synchronization of many individuals in military drill has impressively been described by McNeill (1995).

### Keeping Peace

There is evidence of intra- and intergroup aggression in primates such as chimpanzees (de Waal, 2000), and hominids (Kelly, 2000; Zollikofer et al., 2002; Kelly, 2005). Hominids possessed spears for more than 400,000 years (Thieme, 1997). The advent of tools of potential use as weapons among hominids required even more effective reconciliation means (Wilkins et al., 2012).

To say it with the words of Zollikofer et al. (2002, p. 6447): “The intentional use of implements in the context of intragroup conflict must have had a major impact during hominid evolution because the availability of highly effective hunting and or food-processing tools in interpersonal conflict created a new and considerable potential for intragroup damage, a potential that required specific behavioral adjustments with which to cope. Intragroup aggression in primate societies must be understood as one specific behavioral option in a complex network of social interactions, which is typically balanced by active reconciliatory behavior […].”

This ability is confirmed by the relative scarceness of traces of violence in prehistoric bone findings as compared to skeletons from historic times (Haas and Piscitelli, 2013). Dancing as an effective reconciliatory means has been well-described among potentially hostile Andaman groups by Kelly (2005). Dancing enabled to appease our most dangerous enemies: other men of other tribes or even of the own group (Kelly, 2005; Evans Pim, 2013). Similar to symbolic fights present in many non-human animal species, dance may serve for getting to know who is stronger before undertaking a fight, thus reducing the risk of injury and preventing casualties (Evans Pim, 2013). As an actual example, ghetto dance battles may contribute to avoid deadly duels (McDermott and Hauser, 2005).

### Dance Rituals, Trance, Shamanism, and Religion

(Nietzsche, 1883–1885) argued that he would not believe in any god unless this god was able to dance. Dance in many societies is not only delightment, but it means also to enter into contact with spirits and gods (Métraux, 1958; Lapassade, 1976; Verger, 1982; Ginn, 1990; Christoph and Oberländer, 1996; Jilek, 2009; Herbert, 2011). Although, trance may in some cultures be also reached without dancing, rhythmical techniques including breathing, hyperventilation and dance, as in the Indonesian island of Bali, are the means which are used in the majority of societies for entering trance. Some historical and actual examples for trance dances include the medieval European St. Vitus’ dance, the Italian Tarantella, the Brazilian Candomblé, the Cuban Santería, the Japanese Nô, the Senegalese N’doep or the Sufi Dervish dances. Trance dance serves as catharsis reached through ecstasy. An ancestor, a spirit or a god drives the dancer; the dancer is possessed. Mask dances are common throughout societies worldwide including Malian Dogon, Japanese Kabuki, Dan acrobats in Ivory Coast, Egungun in Benin and Nigeria. Pre-Christian religious mask dances are the origin of present time Carnival traditions. Dancers moving like puppets on the strings such as in Indian Kathakali and Japanese Kabuki are the precursors of theater and pantomime. In this respect, it is interesting that a 15,000-year-old marionette puppet with moveable limbs has been found in a grave of an adult man believed to be a shaman in Brno, Czech Republic (Williams, 2011). In Ethiopian and Greek Orthodox Churches people dance for God. It is still matter of debate whether religion is an adaptive complex itself or a by-product of adaptive behaviors in other non- religious contexts. Since there is no evidence of “natural” non-religious control populations, it cannot be excluded that religious beliefs, at least in hunter-gatherer societies might have provided evolutionary advantages (Boyer, 2001; Dow, 2008; Antweiler, 2015).

### Embodied Pre-verbal Memorizing and Transfer of Traditions

In a pre-verbal context the importance of dance for individual and collective memorizing cannot be overemphasized. Dance in many traditional societies is an instrument to memorize hunting techniques and to preserve traditions by telling stories about the past of the community. In South India Kathakali is danced to tell tales of the Mahabharata epic (Ginn, 1990). Since the mirror motor neurons of who observes dancers are activated dance is an excellent method to train children and adolescents and to communicate experiences and skills which are later internalized by imitation (Rizzolatti et al., 1996). Also in this function, dance is the predecessor of theater (Sachs, 1933/1980).

### Paving the Way for Verbal Communication

Language might have evolved alongside melody, possibly passing through a “musilanguage” stage as already argued by Rousseau (Rousseau, 1781; Brown and Jordania, 2013). However, the evolution of language requires an underlying rhythmic and gestural understanding, i.e., embodied communication (Oberzaucher and Grammer, 2008; Phillips-Silver et al., 2010; Honing, 2012; Gillespie-Lynch et al., 2014). Rhythm perception enables to discern words and is necessary to codify and decode language. The observation of a dancer aids to recapitulate and decode gestures (Patel and Daniele, 2003; Patel, 2008; Hausen et al., 2013; Fujii and Wan, 2014; Magne et al., 2016). Thus, it is likely that R&D paved the way for the evolution of language.

## Individual Fitness

### Individual Psychological Fitness

The individual benefits from R&D in several ways. R&D have anti-depressive effects and divert thoughts from sorrows and boredom. Fetuses are able to hear their mother’s physical functions already from the middle of pregnancy on (Trehub, 2003; Parncutt, 2009; Grahn, 2012). Mother’s breathing and heartbeat may produce an incessant conditioning effect which one could describe as a “soothing fetal brainwash.” Up to here, there is no difference between humans and other mammals. Human babies are, however, especially immature at birth as compared to other animals. Therefore, human infants may require more specific soothing efforts such as rocking. To rock the baby one needs free arms. Soothing a baby by rocking is probably a human universal which, however, has not been investigated in this respect. A recent study comparing cultural effects on rocking a baby for soothing showed more similarities than differences between different cultures (Vinall et al., 2011). There is some research on the effects of rocking in the medical literature. In PubMed we found 157 hits from 1948 to 2014. Especially premature babies benefit from rocking (Malcuit et al., 1988; Clark et al., 1989; Sammon and Darnall, 1994). Rocking has a positive effect on the entrainment of respiration as well as on neuromuscular development of infants (Malcuit et al., 1988; Clark et al., 1989). Intuitively, one may assume that experienced caretakers know that rocking is an effective means to soothe a baby, but if we look very carefully at infants’ behavior we appreciate that the infants themselves induce their caretakers to rock them since other means are less effective. Infants and toddlers exhibit also active physiological stereotypic movements (Sallustro and Atwell, 1978; Thelen, 1979; Barry et al., 2011; Lutz, 2014). Interestingly, physiological rhythmical stereotypies not only have a self-soothing effect as reflected by heart rate reduction but frequently involve the legs, a behavior that could be the starting point of dancing (Soussignan and Koch, 1985). In fact, also later spontaneous unintentional movements to a musical beat most frequently involve the lower limbs reflecting an unconscious proneness to dance (Woods and Miltenberger, 1996; Janata et al., 2012). Whether or not rocking a baby is a behavior strictly confined to humans is an interesting research question which deserves to be explored by evolutionary biologists. We did not find any report of animals or non-human primates rocking their offspring. Moreover, whereas rhesus monkeys have not been found to be good detectors of beat (Honing et al., 2012), chimpanzees display rhythmical behaviors (Ravignani et al., 2013).

It is conceivable that the sensitivity of babies for being rocked and physiological stereotypes paved the way for the evolution of R&D in humans (Soussignan and Koch, 1985). Rocking may also promote the ability of infants to stratify rhythm (Phillips-Silver and Trainor, 2005). Dance enables to self-induce the soothing effect of being rocked. Dance appeases the tormented soul and leads to the secretion of hormones like dopamine and endorphins (Sutoo and Akiyama, 2004; Harris, 2007; Salimpoor et al., 2011; Dunbar et al., 2012). The particularly strong emotional impact of R&D is underscored by recent applications in medicine. Their capacity to influence mood, to reach autistic patients otherwise refractory to any emotional involvement and to make Parkinson patients start moving are taken advantage of in medicine (Hayakawa et al., 2000; Sacks, 2007; See, 2012; Moore, 2013; Nombela et al., 2013; Boehm et al., 2014; Ashoori et al., 2015). Playing musical instruments and dancing reduce the risk of dementia in the elderly (Verghese et al., 2003).

R&D enable to divert the otherwise unstoppable flow of thinking (Steiner, 2006). Dance and music playing enable to psychological “flow” experiences that wipe away unpleasant thoughts, sorrows and boredom (Thomson and Jaque, 2012; Chirico et al., 2015). Boredom may be not only a phenomenon of modern societies but also a problem of traditional societies. Men seem to be more prone to both, boredom and violence, which also are associated with suicide (Wrangham and Peterson, 1996; Heinsohn, 2003). R&D help to overcome boredom and, thus, contribute to keep peace and save lives (Sundberg et al., 1991; Choquet et al., 1993; Wexler and Goodwin, 2006).

R&D are particularly powerful means to express the essential “joie de vivre” (joy of life), i.e., the pure “raison d’être” (reason to exist) a philosophical aspect which has been particularly emphasized by Latin-American and African authors (Giglio and Giglio, 1980; Foix, 2007; Kouam and Mofor, 2011), as Jean Massoulier texted: “Je danse donc je suis” (I dance therefore I am).

### Individual Physical Fitness

Dance, rhythm, music and being rocked have been shown to have painkilling effects (Lehmann, 2010; Pillai Riddell et al., 2011; Dunbar et al., 2012; Johnston et al., 2014). The capacity of music to reduce the dosage of painkilling medication in intensive care patients is documented in medicine (Lehmann, 2010). A recent study has shown that rhythmical music reduces the perceived exertion induced by strenuous physical performance an observation which was well-known to the cotton harvesters in the USA and is reflected by specific working songs. This effect occurs not only on a psychological but also on a proprioceptive level (Fritz et al., 2013). The pain threshold is elevated more by active drumming, dancing or singing than by passive music listening (Dunbar et al., 2012). Rhythmic movements or breathing into hyperventilation are effective means for entering trance, an effect that Hindu yogis take advantage of when they perforate their skin, tongue, or lips before starting their processions.

Furthermore, active and passive rhythmical movements improve body coordination (Trainor and Cirelli, 2015). Although, a major evolutionary advantage is to be expected from cooperation, in some given moments preparation for fighting may be useful for a given group to succeed in winning against enemies and thereby improving the access to resources (Kelly, 2000). Individual and collective coordination skills are trained in martial dances for example in Brazilian Capoeira and Maculelê, in Sicilian Taratatà, Indian Kalaripayattu (Phillips-Silver et al., 2010).

From an evolutionary perspective, all these more or less overlapping aspects are likely to have played a role although these are not equally important at the same time and age. We would tentatively rank reproductive fitness, cooperation and bonding as the driving evolutionary forces whereas the individual aspects may have further contributed to the evolutionary functions of dance in specific age, gender, and prehistoric contexts (Nowak, 2006; Nowak et al., 2010). Survival is particularly important for children in traditional societies with high infant mortality (Carter and Mendis, 2002; Hart and Sussmann, 2009). Reproductive fitness applies to sexually mature individuals who may even risk their lives in order to find potential partners. Peace-keeping and martial dancing could have been particularly important for young men during periods of high violence. On the other hand, martial dances are not human universals and high violence periods have been more widespread in historic times than in prehistory (Kelly, 2000; Haas and Piscitelli, 2013).

In summary, dance offers evolutionary advantages to humans by contributing to sexual reproduction signaling, cooperation, social bonding, infant care, violence avoidance as well as embodied individual and social communication and memorization. Anticipating one consequence of our R&D concept we would expect that not only beat induction is innate but that during their development infants and toddlers spontaneously start to dance earlier than to express other musical utterances such as singing and that this behavior does not depend on the cultural background of their parents. For further investigating the specific functions of R&D in humans, it would be highly interesting to compare the timing of their emergence during the lifespan of humans with the emergence of synchronic behavior in non-human animals.

## Conclusion

The main intention of this article is to provide a refined concept for further interdisciplinary research on the evolution of dance and music in humankind. It is proposed that in future studies on the evolution of music, attention should be paid on which attribute of music precisely is focused whether rhythm, melody, or harmony. The same applies to rhythmical attributes, i.e., pulse of beats, stronger or weaker beats (downbeats, offbeats), tactus, tempo, meter, and patterns. The evolutionary functions of dance have been relatively neglected. The close mutual relationship between rhythm and dance and embodied rhythm perception should be fully acknowledged in future research.

## Author Contributions

JR did the literature search, developed the hypothesis and wrote the manuscript; RO contributed to the literature search, to developing the hypothesis and writing the manuscript.

## Conflict of Interest Statement

The authors declare that the research was conducted in the absence of any commercial or financial relationships that could be construed as a potential conflict of interest.
